# Cranial osteopathy: its fate seems clear

**DOI:** 10.1186/1746-1340-14-10

**Published:** 2006-06-08

**Authors:** Steve E Hartman

**Affiliations:** 1 Department of Anatomy, College of Osteopathic Medicine, University of New England, Biddeford, ME, 04005, USA

## Abstract

**Background:**

According to the original model of cranial osteopathy, intrinsic rhythmic movements of the human brain cause rhythmic fluctuations of cerebrospinal fluid and specific relational changes among dural membranes, cranial bones, and the sacrum. Practitioners believe they can palpably modify parameters of this mechanism to a patient's health advantage.

**Discussion:**

This treatment regime lacks a biologically plausible mechanism, shows no diagnostic reliability, and offers little hope that any direct clinical effect will ever be shown. In spite of almost uniformly negative research findings, "cranial" methods remain popular with many practitioners and patients.

**Summary:**

Until outcome studies show that these techniques produce a direct and positive clinical effect, they should be dropped from all academic curricula; insurance companies should stop paying for them; and patients should invest their time, money, and health elsewhere.

## Background

*"Truth is great, certainly, but considering her greatness, it is curious what a long time she is apt to take about prevailing." *– TH Huxley, 1894 [[1](p218)]

With all I've learned in recent years about human credulity, it remains difficult for me to fathom how little influence fact sometimes has over behavior. For example, 21^st ^century science-based medicine is forced to cope with numerous unfalsifiable (or already falsified) claims from practitioners of the euphemistically labeled "complementary" or "alternative" medical arts, many with names familiar to all: homeopathy, therapeutic touch, reflexology, aromatherapy, magnet therapy...and on, and on, and on. A form of health care of particular interest to readers of this journal which can fairly be labeled "alternative," is cranial osteopathy [2-4]/craniosacral therapy [5]. According to the original biological model [2-4], intrinsic rhythmic movements of the brain (independent of respiratory and cardiovascular rhythms) cause rhythmic fluctuations of cerebrospinal fluid and specific relational changes among dural membranes, cranial bones, and the sacrum. Practitioners believe they can palpably monitor and modify parameters of this mechanism (or a similar mechanism, for example reference [5]) to a patient's health advantage.

## Discussion

Here, focusing on cranial osteopathy, is a cautionary tale inspired by the recent collision of a prescientific, medical reverie with reality in southern Maine.

Once upon a time...

...with the best of intentions, William Garner Sutherland invented cranial osteopathy [2].

Over the years, practitioners convinced themselves that oh-so-gentle palpation of the cranium, guided through understanding of Sutherland's "Primary Respiratory Mechanism," could improve an astounding range of maladies manifesting throughout the human body [6].

Over the years, in both formal (e.g., classroom) and informal (e.g., clinical) settings, ever more students and practitioners learned of Sutherland's (or Upledger's related) [[5](p11)] mechanism and abundant anecdotal success with patients.

Patients were healed, careers were established, and all was good...

...then reality weighed in:

1) As an underlying rationale, the Primary Respiratory Mechanism (including Upledger's "craniosacral" adaptation) [5] has failed utterly:

A. Evidence and biological common sense entirely invalidate Sutherland's mechanism [7,8].

B. Diagnoses based on this mechanism feature not just low reliability but no reliability. There is no evidence, whatsoever, that different practitioners perceive similar phenomena or even that perceived phenomena are real [7,8].

2) After most of a century, no successful, properly controlled outcome analyses have been published. Practitioners have no scientific evidence that their therapeutic actions – however grounded in biology (or metaphysics) – have any direct effect on patient health.

Since 2002, Dr. James Norton and I, together or separately, publicly or privately, and on many occasions have shared our "cranial" skepticism with colleagues around the world, including those at the American Osteopathic Association, the National Board of Osteopathic Medical Examiners (U.S.A.), and the Journal of the American Osteopathic Association. In addition, we have offered our science-based, heavily referenced, critical impressions to readers of *The Scientific Review of Alternative Medicine *(United States) [7,8], *Physical Therapy *(United States) [9], *Ostium *(Australia) [10,11], *The Osteopath *(United Kingdom) [12], *The International Journal of Osteopathic Medicine *(United Kingdom) [13], and in the form of several French translations [14,15]. With many of our publications, letters, E-messages, and personal communications, we have invited practitioners to inform us of scientific work we may have missed or misinterpreted. Knowledge of such might prompt us to reconsider our negative conclusions regarding the biological mechanism, diagnostic reliability, and clinical efficacy of cranial osteopathy/craniosacral therapy. After four years since our first joint publication, we remain unaware of published, substantive rebuttals or of work suggesting that our views should be refined in any way.

The End?

Well, it should be but it's not. The therapeutic ministrations of many "cranial" practitioners derive directly from the now invalidated, anomalous Primary Respiratory Mechanism. This means that up-to-date practitioners no longer have even the imaginary biology of Sutherland's mechanism to explain what they do or why they believe it works. Some clinicians of my own college of osteopathic medicine disavow intellectual allegiance to the mechanism but cling to it as a "teaching metaphor"...because they otherwise lack even this failed biological device to unify and explain their diagnostic and therapeutic propositions. Some counter criticisms by changing the subject to the perceived array of poorly understood conventional treatments. Many deflect criticism by focusing, instead, on their perceived (but scientifically almost meaningless) personal clinical success. Many practitioners around the world disown Sutherland's biology-based mechanism altogether (or were trained in a somewhat different model) and instead engage objectively immeasurable body energies [[16](p169–170), [17](p144–147), [18-21]], quantum mechanics [[16](p55–56), [17](p137–138), [19]], vitalism [[17](p141–147), [19,22](p14–16), [23]], or God [[16](p123–124)].

So the Primary Respiratory Mechanism is gone and there is no evidence of efficacy...but cranial osteopathy/craniosacral therapy, as a belief system, soldiers on. What could be, at most, a placebo, is taught – as medicine – in all colleges of osteopathic medicine in the U.S. [3], is tested for – as medicine – on osteopathic licensing examinations in the U.S. [13], and is practiced – as medicine – around the U.S. and abroad. Practitioners of the "cranial" arts all may be caring, otherwise competent physicians – and some are close friends – but they have hitched their professional wagons to a fantasy and are understandably reluctant to disengage.

As a scientist in this age of evidence-based practice, I have grown frustrated in my dealings with the "cranial" faithful. As a group, evidence carries little weight with them. In our own professional community, skepticism has drawn rebuke and charges of disloyalty, rather than reasoned debate – but I was not surprised. Early in my study I concluded that cranial osteopathy is a pseudoscientific belief system, maintained – by both patients and practitioners – through operation of well- and widely understood principles of human personal and social psychology. From that standpoint, practitioners simply have defended passionately held views to which they long have been committed. Cognitive dissonance [24] inspired by our disbelief brought exactly the reaction we anticipated. Although I remain hopeful that practitioners and healthcare disciplines wedded to these techniques – especially osteopathy – soon will let evidence guide policy, responsible action will not come without trauma. Cranial osteopathy has so long maintained its place in the osteopathic fabric that great personal and political courage now will be required to remove it.

## Summary

After millennia as socially sanctioned, organized magical thinking, medicine has become a powerful service profession. This transition was possible only because scientific inquiry has become integral to almost everything physicians do. Without science, medicine would still involve little more than applying tourniquets, setting bones, and administering placebos. Cranial osteopathy/craniosacral therapy is not a medicine for this century. Perhaps properly controlled outcome studies will show that, though biologically anomalous, these techniques nonetheless produce a direct and positive effect on patient health. Until they do, however, the "cranial" arts should be dropped from all academic curricula; insurance companies should stop paying for them; and patients should invest their time, money, and health in treatments grounded in the extraordinarily successful, science-based biomedical model of the modern era.

## Competing interests

I have taught at the same college of osteopathic medicine for 20 years. Ordinarily, this might prompt suspicion that I have not been openly forthcoming in my criticism of osteopathy's "cranial" subdiscipline. To the contrary, some members of my professional community have questioned my loyalty, apparently believing that my views might have a negative impact on the college or the osteopathic profession. Otherwise, I declare that I have no competing interests.
